# Extending the Field of Dentistry

**Published:** 1910-01-15

**Authors:** 


### EXTENDING THE FIELD OF DENTISTRY.

BY ICONOCLAST.

In "Dental Brief" for December.

After the appearance of three papers of this series it occurs to the writer that it would be well to review the several articles which have been contributed in the way of comment. But first a few general observations may not be deemed inappropriate.

The chief foe to progress is that state of mind which assumes that "whatever is, is right." We form certain opinions, the result of our instruction and environment, and measure everything by those standards. But an open mind, coupled with trained judgment and sane reasoning, is the chief agent in the world's progress. It is our right to disagree with the innovator, while respecting his motives and applauding his investigating spirit.

There are so many subjects upon which we must pass judgment that none of us may hope to be right always but that must not stifle the voice of progress nor stay the feet of the explorer. What the world needs more than anything else is to think, and whoever inspires thought and discussion is among the worthy. The threshing out of ideas is the world's most constant and important duty.

In all discussions where the object is something more than personal or partisan advantage we must exercise care not to be governed by terms, phrases and words. We desire to get at the essence of things, and must not be disturbed if our friends fail to appreciate our distinctions, but with undue haste seek to crowd us into a pigeon-hole labeled "unethical" or "ethical," "advertiser" or "benefactor." We shall refuse to stay in any pigeon-hole, reposing under our label, unless it appears to be both comfortable and appropriate.

To clear up certain possible misconceptions the statement may be made that dentistry has made wonderful progress. With all the criticism heaped upon individuals who degrade their profession, dentistry is honored and is intitled to honor. We have our quacks; but so does the medical profession, and so do the legal and clerical professions. The fact is that it takes men to make dentists, doctors and lawyers and clergymen, and men come in bewildering variety. No profession will ever be distinguished for its absence of quacks or its preponderance of angels. So there is no need of beating the air over such matters. But possibly there is need of considering the general professional outlook, with the object of advancing our standards in keeping with modern requirements and our professional necessities. And this requires that we discuss matters freely and without timidity.

Now let's get our bearings. Some persons seem perturbed lest we give comfort to the "advertising" dentist and they assure us that all dental advertisers are quacks and that there never came from their offices a decent piece of work. That would assume that we are engaged in the effort to destroy the profession rather than striving to advance it; and we wish to be credited with average intelligence. Then let us lay down as a fundamental proposition that if our profession, or any profession, is to advance it must be on the basis of sheer merit. Permanently its measure of honors and rewards will be the measure of its service to humanity. Fraud and quackery do not succeed permanently anywhere, either in businessor inthe profession. Suppose, then, that all dentists were to turn advertising quacks and flood the public with literature and crowd their offices with victims, what would be the result? Why, in five years dentistry would go back to a simple matter of extracting teeth and as a profession would be deader than Hector.

Manifestly, that is not our proposition. There is something else. We have a feeling that many members of the profession have the "advertising" dentists on the brain If the "dental parlor" man succeeds we rave and predict his ultimate failure. Then, if he doesn't fail, we hurl at his head a variety of epithets that show considerable ingenuity as well as perturbation. Do we imagine that we stop the "dental parlor" habit by fulminations of this sort? Would it not be well to get down and calmly inquire why men establish these "parlors," why they advertise and why they generally succeed? If they are all quacks, never do an honest piece of work, prey upon the public, charge outlandish prices and merely suck the life blood of their dupes, how is it that they can still flourish and not be found out? Now that question cannot be answered by an epithet. To withdraw and wrap one's self in the mantle of ethics doesn't settle it. Silence doesn't furnish a solution. There are several problems like this that may lead to a great advance if we study them calmly, admit the facts and try to ascertain the line of action they suggest.

In commenting on these several papers published in the September and October numbers of *The Brief*, it seems unnecessary to state that those who agree with our line of thought have added much of value to the discussion, yet require no comment in return; while those who take issue with us naturally compel us to restate or defend our position.

Doctor Zederbaum, in a friendly way, indicates a number of points of agreement with our views, but notes certain exceptions. For instance, he cannot see that dentists are "a supersensitive aggregation," and thinks that the press tends to belittle the profession with such headlines as "Tooth Carpenters," "Tooth Pluggers' Convention," "Forceps Wielders in Session," etc. But we must remember that oftentimes real epithets are used as terms of respect and even affection. Perhaps you would rather your son call you "Dad" than the dignified term of father. There is no real lack of respect for the President when we read that "Big Bill Taft" visited our city, or that "Teddy the Terrible" has bagged another lion. As a matter of fact, are we not on the watch for some slight to our professional dignity, and wouldn't we be wiser to ignore such trifling incidents and correct any erroneous impressions of our place in the world by enlarging the sphere of our usefulness, thus commanding a larger measure of respect?

This writer also suggests, for the further enlightenment of the profession, the giving of illustrated lectures, "doing it in an informal manner, the same as you would before an audience of workingmen and their families.

This suggests to our mind, Why not actually give them to workingmen and their families? Do we not assume that dentistry is a matter for dentists; that they, as parties of the first part, alone are interested? But where do the parties of the second part come in? They are expected to know when their teeth need attention and to come to us when necessary. That requires considerable information; yet the parties of the first part hold a convention, have lantern slides and discuss dentistry in simple terms, and bar out those workingmen and their families. Why? Would it hurt anything—even our professional dignity—to invite them in? It is well to insist on educated and skilled dentists, but don't we need a people educated in the matter of attention to their teeth, to know when to have work done and, in general, how it should be done? We rail at the quacks, but do they not live on the credulity of the ignorant—those whom we think it unprofessional to help educate.

Dr. Taylor raises a point that the dentist should be a thoroughly educated man; yet Dr. Taylor says that "our schools are clamoring for a higher entrance standard. The students with a better education in language are usually graduated, while the better manipulator, and oftentimes the better reasoner, is turned down."

Dr. McKay, in his interesting article in the October *Brief*, in discussing the necessity for intelligent publicity says: "One important place to do this before all other places is beside our own dental chair; in our own citadel, so to speak."

That covers a great part of the question. The patient hasn't come to you to talk about the weather or the opera. If it be a woman, she is just now interested in the subject of teeth. The opportunity for imparting dental information is made for you. You can tell her an interesting and truthful story that will be to her advantage and yours. You can give her the history of her own teeth, what neglect has done and will do, and the relation of dental health to general physical health.

You can and should go further. You are not employed merely to fill a tooth or set a crown; you are her professional adviser and can with perfect propriety discuss with her the importance of having her children's teeth properly cared for and explain the work in detail. To be a clam is not to be ethical; and if the dentist is himself not profoundly interested in such matters he should have been a farmer or a tinsmith rather than a dentist.

Take a hint from the family doctor. A mother calls, requiring medical attention. After the prescription has been given the doctor says:

"How is Willie?"

"Why, doctor, he doesn't seem to improve; he has little appetite and continues to sleep with his mouth open."

"Well," says the doctor, "we will have to remove those adenoids. Better attend to it at once. Bring him in next Tuesday and I'll fix him up."

Then he asks, "How is Mary's cold?"

"She has that cough yet, doctor."

"That won't do; we must stop it. Bring her in some day this week and I'll go over her and see if I can't do something for it."

Is that doctor unethical? Has he any lesson for the dentist?

There are several points in Dr. Taylor's paper that invite discussion. A young man was once asked what he filled teeth for. And his answer was, "To get money."

Exactly! The young man was honest. If he had said that he filled teeth merely to benefit humanity he would have been a prevaricator.

Men preach to get money; they practice medicine to get money. A man who will not contrive to support himself and his family is a bad egg; and to support them he must make money. If he wishes to make it honestly, to give value received, then the more he makes the more he gives.

But while we all wish to make money, that isn't *all* we wish to do. We may still have our higher ideals, our charitable impulses, and these will mingle in our practice, relieving it of the stigma of being "dollar-getting."

Dr. Taylor's article brings up another question—the "Dental Parlor." He asks: "Did anybody ever get a mouth put in good order in a so-called dental parlor?"

Yes; excellent work has been done in dental parlors. But this is not a defense of dental parlors or of individual advertising. Our proposition is that *dentistry* needs more intelligent publicity; that the individual dentist can disseminate useful information regarding the care of the teeth and can with perfect propriety, as Dr. Taylor says, teach well while working at the chair.

And here is food for a little thought: If every dentist would do good, faithful teaching while working at his chair; if he would embrace every opportunity to make known what his profession has to offer humanity, would the advertising dentist then have the same inducements to advertise, and would his financial rewards encourage him to continue? In other words, has not the attitude of the ethical dentist made the opportunity for the advertising man? Nothing will put the advertiser out of business but competition. But what kind of competition? Not his own kind, but a different sort.

Dr. Wilson says the field is "limited and should be more so." He shows conclusively that the profession has made wonderful progress. He says that at the close of the Revolution we had one dentist for nearly 3,000,000, while we have now one dentist for every 3,500 people. This is a very good estimate; but if we allow to each dentist 300 patients who have their teeth regularly and systematically cared for, we have about 70,000,000 persons who visit a dentist either seldom or never at all.

Can the profession extend its services to these 70,000,000 of people? Can we interest and educate them? Can we make any progress along this line whatever, or shall we give up the task as hopeless? And if we give it up, what will become of the proud boast of the ethical that their mission is "the service of humanity?"

Dr. Kent thinks that space should be given in our dental journals to "words of wisdom and advice from good men on the commercial side of dental practice." Without objection let us substitute "business" for "commercial" since all will admit that every profession, including the ministry, has its business side.

It is a good suggestion, since the most successful dentists lay *all* the stress upon their professional attainments, when in all probability their success is in part due to the fact that they are good business men. Let them frankly tell their brother practitioners what they know in this department.

This brings us to Dr. Beadle's paper. He thinks we should decide whether we belong to a profession or to trade. There is no question but that dentistry is a profession; and there is no question but that every profession has its business side. We must not be governed by words and shape our lives to meet the requirements of a phrase.

But even though we drop out of consideration the business or "trade" idea, as *professional* men are we not under obligation to extend the field of our usefulness to the masses? As a matter of fact, are we not giving our attention to the few who are willing to pay for it while holding up the ideal of service "to humanity?" Is it wrong for the profession generally to more fully educate the great public in the necessity of the care of its teeth? If anybody objects to the selfishness of this object, in case it increases his practice he has the option of turning a part of it over to some less fortunate brother who may need it.

There is an honest note in the paper of Dr. Kent, which is condensed in this clean-cut sentence:

"He hears the call of suffering humanity, but sees no reason why it should deafen him to the call of his household."

And, let us add, whoever does not take care of himself and family is not apt to do much for humanity. This is not an age of holy pilgrimages, and the world should be thankful that it is not.

